# Effects of Selenium-Enriched Protein from *Ganoderma lucidum* on the Levels of IL-1****β**** and TNF-****α****, Oxidative Stress, and NF-****κ****B Activation in Ovalbumin-Induced Asthmatic Mice

**DOI:** 10.1155/2014/182817

**Published:** 2014-02-10

**Authors:** Guan Min-chang, Tang Wei-hong, Xu Zhen, Sun Jie

**Affiliations:** ^1^Taizhou Enze Medical Center Luqiao Hospital, Zhejiang 318050, China; ^2^Huzhou Central Hospital, Zhejiang 313000, China

## Abstract

The purpose of this study was to compare the effect and toxicity of organic selenium (Pro-Se) with inorganic selenium (IOSe) in preventing asthma in ovalbumin-induced asthmatic mice. After the mice were treated orally with Pro-Se and IOSe, respectively, the plasma Se levels, Se accumulation in liver and kidney, tumor necrosis factor alpha (TNF-**α**), interleukin 1 beta (IL-1**β**), oxidative stress, and NF-**κ**B activation in lung were examined. The results showed that the serumal Se levels in the mice fed the Pro-Se were significant (*P* < 0.01) elevations. It results in restoration of the level of endogenous antioxidant enzyme, lower levels of TNF-**α** and IL-1**β**, and activated NF-**κ**B in the asthmatic mice. Our experiments have demonstrated profound differences between the activities of organic selenium and inorganic selenium in experimental conditions. These data provide an important proof of the concept that organic selenium might be a new potential therapy for the management of childhood asthma in humans.

## 1. Introduction

Asthma is a chronic disease associated with airway hyperresponsiveness, airway obstruction, and airway remodelling [1, 2]. The principal pathophysiology of asthma is chronic inflammation of the lower respiratory tract [3]. Anti-inflammatory agents such as inhaled steroids and leukotriene receptor antagonists along with long acting bronchodilators are the mainstay of asthma pharmacotherapy. However, potential long term side effects, prohibitive costs, and suboptimal adherence to asthma medications are ongoing challenges to optimal asthma control. Treatment options are, therefore, quite limited for asthma and the need to search for other therapies has been recognized by many experts in the field [4, 5].

The trace element selenium (Se) is an essential nutrient for all mammalian species and is of fundamental importance for human biology. Deficient Se intake may dramatically affect inflammation and immune responses. Also, the use of Se supplementation to increase Se status to supraphysiological levels may be exploited to modulate immune processes that drive certain health disorders, such as the T helper 2 (Th2) responses that drive allergic asthma [6, 7]. Therefore, Se intake has been hypothesized to play a role in the development and/or severity of this complex disease, asthma.

However, Mahan and Kim [8] suggested that Se may not be as biologically effective as the Se indigenous in grains, which is primarily in the organic form of selenomethionine. Mahan and Parrett [9] found that total Se excretion decreased and tissue retention increased when an organic, rather than an inorganic, source of Se was fed. Further, organic selenium is reported to be better absorbed, has higher bioavailability, and is less toxic than inorganic selenium [10–12]. In the present study, we isolated selenium-contained protein (Pro-Se) with different selenium content, indicated the organic selenium, from the *Ganoderma lucidum*, and compared the effect and toxicity of organic selenium in preventing asthma with those of inorganic selenium (IOSe).

## 2. Materials and Methods 

### 2.1. Chemicals

Sodium selenite was purchased from Anhui Star New Material Technology Co., Ltd., China.

### 2.2. Sodium Selenite Solution (IOSe)

Sodium selenite was dissolved in normal saline. An ampule was filled with 0.4 mL of IOSe with 0.104 *μ*g sodium selenite.

### 2.3. Selenium-Contained Protein (Pro-Se)

*Ganoderma lucidum* was cultured on the medium which was composed of peptone (10.0 g/L), beef extract (10.0 g/L), yeast extract (5.0 g/L), K_2_HPO_4_ (2.0 g/L), triammonium citrate (2.0 g/L), sodium acetate (5.0 g/L), glucose, tween 80 (1.0 mL/L), MgSO_4_7H_2_O (0.58 g/L), MnSO_4_H_2_O (0.25 g/L), corn steep liquor (3.0 g/L), and cysteine hydrochloride (0.3 g/L). After 6 h, sodium selenite of different concentrations (0, 2.5, 5.0 mg/mL) was added to the medium. The Se-enriched* Ganoderma lucidum* were marked with Se0, Se2.5, and Se5.0, respectively, according to the sodium selenite concentration. The Se-enriched *Ganoderma lucidum* was removed by centrifugation after cultured with sodium selenite for another 12 h, and then it was preserved in the dark at room temperature after freeze drying (−50°C, under vacuum).

The protein was isolated according to the method of Kadrabova et al. [13]. 3.0 g of each kind of dry bacteria samples (Se0, Se2.5, and Se5.0) was dissolved in cold sodium hydroxide solution (0.25 M); the bacteria samples were broken by ultrasonication and incubated at 50°C in water bath for 2 h. The supernatant was obtained by filtration and the residue was repeated twice with 50 mL of NaOH (0.25 M). Then, ammonium sulfate was added to the supernatant to make 95% saturated solution which was followed by keeping it overnight at 4°C. Protein was precipitated using centrifuge at 6000 rpm for 30 min at 4°C. The resulting precipitate was then dissolved in 10.0 mL of Tris-HCl (pH 8.0, 50 mM). This solution was passed through 0.22 mm syringe filter and dialysed against 1.0 L of Tris-HCl (pH 8.0, 50 mM) using a membrane with 3500 molecular weight cutoff at 4°C three times to remove ammonium sulfate. Each kind of protein was marked with Pro-Se0, Pro-Se2.5, and Pro-Se5.0. The selenium content in each protein sample has been determined to be 0, 0.260, and 0.333 mg/L, respectively, by hydride generation-atomic absorption spectrometry.

### 2.4. Animals

Twenty-to-22-day-old female BALB/c mice, weighing from 12 g to 15 g, were obtained from the Experimental Animal Center of Zhejiang. Mice were housed with free access to food and water in a room with an ambient temperature of 22 ± 2°C and a 12 : 12 h light/dark cycle. All experiments were carried out in strict accordance with the National Institutes of Health Guide for the Care and Use of Laboratory Animals. The normal basal diet (NBD) for the mice was also purchased from the Center of Laboratory Animals, Zhejiang University. The NBD containing 0.05 *μ*g/g of Se in a pellet form was formulated to meet the nutrient requirements for normal laboratory mice.

### 2.5. Induction of Model of Asthma

Mice were sensitized via 2 intraperitoneal injections of 10 *μ*g of ovalbumin (grade V, ≥98% pure, Sigma, St. Louis, MO, USA) with alum adjuvant on days 0 and 14 of the experiment. Starting on day 21, the mice, housed in whole-body exposure chambers, were exposed to 1% aerosolized ovalbumin for 30 min a day, 3 days a week, for 9 weeks. The temperature was kept at 20°C to 25°C and the relative humidity at 40% to 60%.

### 2.6. Experimental Design

Mice were randomly assigned to six groups: control group with saline treatment, asthmatic group with saline treatment, and asthmatic group treated with 0.4 mL Pro-Se0 (0 *μ*g selenium), 0.4 mL Pro-Se2.5 (0.104 *μ*g selenium), 0.4 mL Pro-Se5.0 (0.1332 *μ*g selenium), and 0.4 mL of IOSe (0.104 *μ*g selenium). All animals surviving after 30 days were killed. The blood samples were drawn from orbital vein from all the groups and serum was separated for biochemical estimations. Tissue samples of lung, liver, and kidney were dissected from the visceral tissues. After washing with saline, the tissue samples were blotted dry and weighed. All samples were stored at −80°C for future analysis.

### 2.7. Serum Selenium Measurement

Plasma Se levels were determined by means of an atomic absorption spectrometer (AAS-3200, Shanghai, China). Plasma was diluted (1 : 3) with 0.05% Triton-X 100 and 0.05% antifoam B solution. NiNO_3_ (0.2% w/w) was used as a modifier for Se measurement. All determinations were run in duplicate, and individual values were averaged. The highest standard addition concentration was 100 *μ*g/L for Se determination. The autosampler system was used in the automix mode and with sample intake of the graphite tube of 1 *μ*L/s. The total volume inserted in the tube was 20 *μ*L (5 *μ*L of matrix modifier, 10 *μ*L of sample, and 5 *μ*L of standard solution). Absorption readings were measured as peak height. The variation coefficient for replicate measurement was <3%. The lowest threshold was 10 *μ*g/L for Se detection.

### 2.8. Determination of Se Accumulation in Liver and Kidney

Selenium concentrations were determined using atomic absorption (AA). Weighed aliquots of frozen tissues were digested in 3 stages: the first using 5 mL mixed acids (4 : 1 of nitric acid : perchloric acid), the second using a combination of 2 mL HNO_3_ and 30% H_2_O_2_, and finally using 2 mL HNO_3_. All digestions were performed at 130°C until the acid was completely evaporated and the residue dried before the next acid stage was started. After the third acid treatment, 1% HNO_3_ was added to the digests and heated at 80°C for 1 h. After cooling, the sample volume was measured and analyzed.

### 2.9. Estimation of Pro-Se on Oxidative Stress

Serum was used for the assay of glutathione (GSH) content, Lipid peroxidation (LPO), glutathione peroxidase (GPx) activity, glutathione reductase (GR) activity, catalase (CAT) activity, Na^+^K^+^ATPase activity, and glutathione S transferase (GST) activity.

### 2.10. Measurement of Pro-Se on IL-1*β* and TNF-*α*

The concentration of interleukin-1*β* (IL-1*β*) and TNF-*α* in the plasma was determined using a commercial ELISA kit (Shanghai Jinma Biological Technology Inc., China) according to the manufacturer's instructions.

### 2.11. Measurement of Pro-Se on NF-*κ*B Activation in Lung

For measurement of NF-*κ*B activation in the lung, nuclear protein from lung samples was extracted using a commercial kit (Shanghai Jinma Biological Technology, Inc., China). Activation of NF-*κ*B in the lung was determined using an ELISA kit (Shanghai Jinma Biological Technology, Inc., China), following the manufacture's instruction. This kit specifically detects the p50 member of NF-*κ*B.

### 2.12. Data Analysis

Group means were compared by ANOVA with GraphPad Prism (GraphPad Software, Inc.). Multiple comparison tests were performed with Tukey for significant differences at *P* < 0.05.

## 3. Results

### 3.1. The Effect of Pro-Se on Selenium Levels in Serum

As shown in Table 1, Pro-Se significantly increased Se content in mice serum. Compared to the control without diet supplementation, serum Se levels were improved by 10.4% (*P* > 0.05), 27.1% (*P* < 0.05), and 64.6% (*P* < 0.01) with IOSe, Pro-Se2.5, and Pro-Se5.0, respectively. Pro-Se was more favorable to Se absorption and deposition in rats than IOSe, which indicated that the Pro-Se in this study showed a higher bioavailability in mice.

### 3.2. Se Accumulation in Liver and Kidney

**Figure 1:**
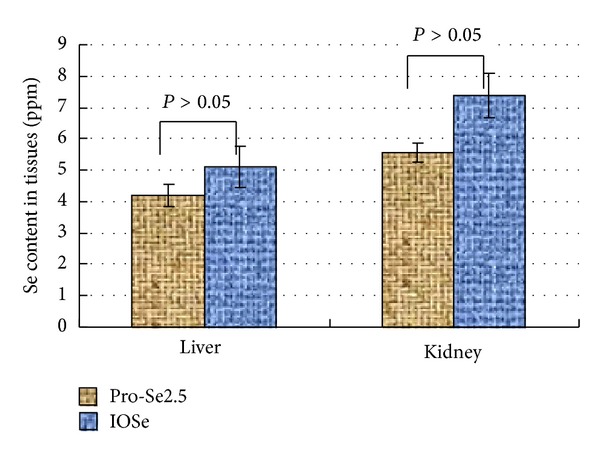
Se contents in animal tissues. The Se content in tissues was not significantly different between Pro-Se and IOSe groups (*P* > 0.05).

The organ masses of liver and kidney were significantly different between Pro-Se and IOSe groups (*P* < 0.05). However, there was no significant difference in organ masses of liver and Kidney between the Pro-Se groups (*P* > 0.05). It is implied that IOSe is toxic to animals, while Pro-Se is essentially nontoxic (Table 2). The Se contents in animal tissues were measured following the studies and the results are shown in Figure 1. The Se content in tissues of Pro-Se2.5 group was 4.21 ppm and 5.56 ppm (liver and kidney) while IOSe groups exhibited elevated levels of Se content, but not to a statistically significant degree. It is implied that Pro-Se could reduce Se accumulation in animal tissues to a certain extent.

### 3.3. The Effect of Pro-Se on Oxidative Stress

In this study, a significant increase in the activity of LPO was observed in asthmatic group, as compared to the control group (*P* < 0.001), whereas Pro-Se2.5 and Pro-Se5.0 treatment significantly (*P* < 0.05, *P* < 0.01, resp.) resulted in decreased LPO levels when compared with asthmatic group (Table 3). Concentrations of GSH were lower in asthmatic group than those in control group (Table 3). Pro-Se produced the increase in the level of GSH. The activity of endogenous antioxidant enzymes was decreased significantly (*P* < 0.01) in the asthmatic group, as compared to the control group, whereas in the Pro-Se2.5 and Pro-Se5.0 groups, Pro-Se treatment showed a significant (*P* < 0.05–0.01) restoration in the level of various enzymes as compared with asthmatic group (Table 3). However, there was no significant difference between the asthmatic group and the IOSe treated group (Table 3).

### 3.4. The Effect of Pro-Se on Inflammatory Mediators

**Figure 2:**
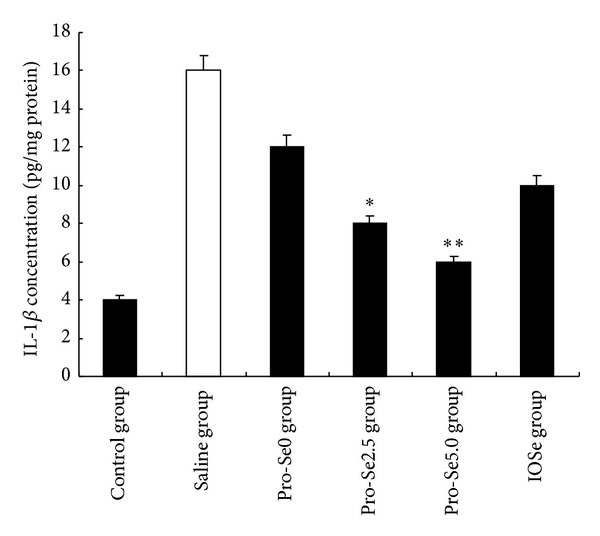
Effect of Pro-Se and IOSe on IL-1*β* concentration. Values are shown as means ± SEM. *P < 0.05 versus asthmatic group.

**Figure 3:**
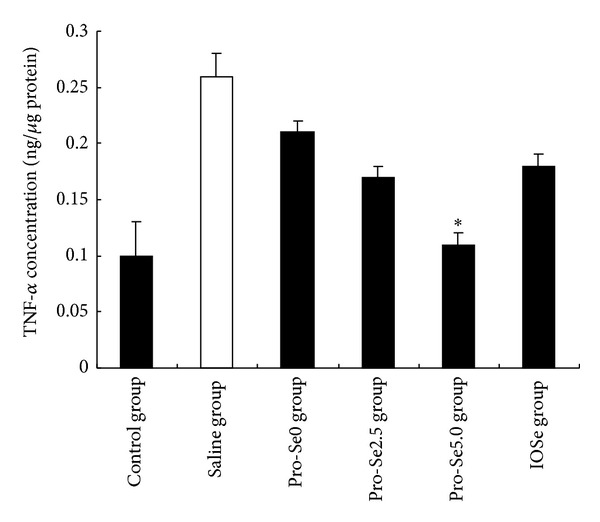
Effect of Pro-Se and IOSe on TNF-*α* concentration. Values are shown as means ± SEM. *P < 0.05 versus asthmatic group.

Figure 2 shows that ovalbumin-induced asthma significantly increased protein concentration of IL-1*β* in the blood. Pro-Se2.5 and Pro-Se5.0 treatment decreased the level of IL-1*β* as compared to the asthmatic group, respectively (*P* < 0.01, *P* < 0.05). As shown in Figure 3, the levels of TNF-*α* elevated significantly after ovalbumin-induced asthma. Pro-Se5.0 suppressed this response (*P* < 0.05). However the same result did not occur in the IOSe treated group.

### 3.5. The Effect of Pro-Se on Activation of NF-*κ*B

**Figure 4:**
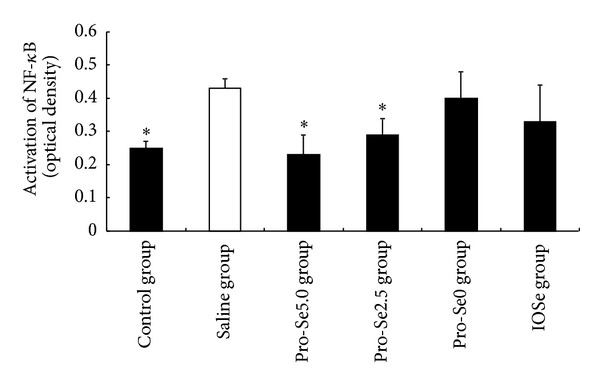
Effect of Pro-Se and IOSe on activation of NF-*κ*B. Values are shown as means ± SEM. *P < 0.05 versus asthmatic group.

As shown in Figure 4, asthma significantly induced activated NF-*κ*B above control levels, and as hypothesized, Pro-Se2.5 and Pro-Se5.0 significantly suppressed this response. However, the same results did not occur in the Pro-Se0 treated group and IOSe treated group.

## 4. Discussion

It has been reported that selenium supplementation might be beneficial to patients with intrinsic asthma [13]. However, the less absorbed and bioavailability limits its role as a therapeutic agent for asthma [10–12]. In the present study, we isolated Pro-Se from* Ganoderma lucidum* and compared the difference of the antioxidant and anti-inflammatory activities between organic selenium (Pro-Se) and inorganic selenium (IOSe) in the model of asthma.

In this study, we have demonstrated a correlation between Se and asthma. Asthma led to a decrease in serum Se level. In our *in vivo* study, we have presented evidence for a significant increase in serum Se level in Pro-Se treated asthmatic mice while IOSe did not exhibit any such effects. It indicates Pro-Se may improve the bioavailability of Se in mice. At the same time, the Se content in tissues of Pro-Se2.5 group was lower than those of IOSe groups. It is implied that Pro-Se could reduce Se accumulation in animal tissues to a certain extent.

The lungs of asthmatic patients are exposed to oxidative stress due to the generation of reactive oxygen as a consequence of chronic airway inflammation. The available evidence tends to support the concept that the oxidant/antioxidant equilibrium is disturbed in asthma [14, 15]. Therefore, the measurement of endogenous antioxidants enzymes, that is, GPx, GR, CAT, and GST as well as Na^+^K^+^ATPase, has been performed to estimate the amount of oxidative stress. Se plays an important role in the antioxidant defence system [16, 17]. In the present study, asthmatic mice had lower levels of antioxidant enzymes such as GST, GPx, CAT, GR, and catalase activities, whereas in the Se group, Pro-Se treatment showed a significant restoration in the level of various enzymes as compared with asthmatic group. The same results did occur in the IOSe group. These findings are consistent with the presented evidence for a significant increase in serum Se level in Pro-Se treated asthmatic mice.

Adequate assessment of inflammatory cells, cytokines, chemokines, and anti-inflammatory molecules is essential for understanding, monitoring, and treating lung diseases. Among these inflammatory mediators, IL-1*β* and TNF-*α* are of particular importance because they play a major role in coordinating mechanisms that command proinflammation. The suppression of these proinflammatory mediators has been found to reduce the severity of the inflammatory reaction [18]. We found that Pro-Se significantly reduced the levels of IL-1*β* and TNF-*α*. However, the same result did not occur in the IOSe treated group. These studies support our hypothesis that Pro-Se may enhance Se anti-inflammatory effects.

NF-*κ*B activation is correlated with significant increases in IL-1*β* and TNF-*α* mRNA levels [19]. Therefore, we hypothesized that Pro-Se may potentially show beneficial effects by decreasing the expression of NF-*κ*B. As shown in Figure 4, NF-*κ*B expression in the saline group was significantly higher than that in the control group (*P* < 0.01), and Pro-Se treatment significantly suppressed asthma-induced NF-*κ*B expression. These results suggest that the inhibitory effects of Pro-Se on expression of the NF-*κ*B p50 subunit are associated with increase in serum Se level in Pro-Se treated asthmatic mice.

In conclusion, this study clearly shows that organic selenium (Pro-Se) is able to counteract the asthma in an experimental model by using a variety of testing systems. Also, our experiments have demonstrated profound differences between the activities of organic selenium and inorganic selenium in experimental conditions. These data provide an important proof of the concept that organic selenium might be a new potential therapy for the management of asthma in humans. The potential application of organic selenium needs to be further studied.

## Figures and Tables

**Table 1:** The effect of Pro-Se and IOSe on selenium levels in serum (*n* = 10).

Different groups	Selenium level (*μ*g/mL)
Asthmatic group with saline	0.48 ± 0.03
Asthmatic group with Pro-Se5.0	0.79 ± 0.09**
Asthmatic group with Pro-Se2.5	0.61 ± 0.01*
Asthmatic group with Pro-Se0	0.34 ± 0.06
Asthmatic group with IOSe	0.53 ± 0.06
Control group	0.72 ± 0.03

*P < 0.05, **P < 0.01 versus saline-treated.

**Table 2:** Effects of Pro-Se and IOSe on organ masses of mice (*n* = 10).

Mouse group	Liver weights (g)	Kidney weights (g)
Control group	1.47 ± 0.05^a^	0.49 ± 0.04^a^
Asthmatic group with saline	1.30 ± 0.07^b^	0.37 ± 0.03^b^
Asthmatic group with IOSe	1.31 ± 0.06^b^	0.34 ± 0.03^b^
Asthmatic group with Pro-Se0	1.48 ± 0.07^a^	0.48 ± 0.04^a^
Asthmatic group with Pro-Se2.5	1.47 ± 0.03^a^	0.47 ± 0.02^a^
Asthmatic group with Pro-Se5.0	1.45 ± 0.06^a^	0.49 ± 0.02^a^

The different letters in the same column indicate a statistical difference (*P* < 0.05).

**Table 3:** Effect of Pro-Se and IOSe on GSH and LPO levels and the activity of various enzymes.

Different groups	GSH (nmol/mL)	LPO (nmol/mL)	GPx	GR	GST	CAT	Na^+^K^+^ATPase
Control group	1.830 ± 0.011*	14.33 ± 0.56***	16.00 ± 2.23**	36.56 ± 2.56**	17.44 ± 1.23**	7.22 ± 0.32**	4.56 ± 0.60*
Asthmatic group with saline	1.112 ± 0.011	20.01 ± 1.41	7.89 ± 0.33	21.11 ± 2.23	9.07 ± 1.11	4.66 ± 0.10	2.22 ± 0.20
Asthmatic group with IOSe	1.401 ± 0.022*	19.82 ± 1.22	8.10 ± 0.32	24.31 ± 2.02	10.60 ± 0.66	4.88 ± 0.32	3.00 ± 0.31
Asthmatic group with Pro-Se0	1.300 ± 0.011*	17.80 ± 1.11	6.12 ± 0.30	20.31 ± 2.00	9.60 ± 0.33	3.80 ± 0.25	2.00 ± 0.30
Asthmatic group with Pro-Se2.5	1.500 ± 0.021*	17.23 ± 2.00*	13.23 ± 1.30*	27.50 ± 2.11**	15.50 ± 1.15*	5.45 ± 0.55*	4.32 ± 0.30*
Asthmatic group with Pro-Se5.0	1.500 ± 0.033*	14.33 ± 3.21*	14.11 ± 1.12**	30.21 ± 6.03**	17.66 ± 2.33**	6.66 ± 0.44*	4.11 ± 0.21*

Values are shown as means ± SEM. *P < 0.05 versus asthmatic group, **P < 0.01 versus asthmatic group, and ***P < 0.001 versus asthmatic group.

## References

[1] Agrawal DK, Shao Z (2010). Pathogenesis of allergic airway inflammation. *Current Allergy and Asthma Reports*.

[2] McGee HS, Stallworth AL, Agrawal T, Shao Z, Lorence L, Agrawal DK (2010). Fms-like tyrosine kinase 3 ligand decreases T helper type 17 cells and suppressors of cytokine signaling proteins in the lung of house dust mite-sensitized and -challenged mice. *American Journal of Respiratory Cell and Molecular Biology*.

[3] Hargreave FE, Nair P (2009). The definition and diagnosis of Asthma. *Clinical and Experimental Allergy*.

[4] Chakir J, Hamid Q, Bossé M, Boulet L-P, Laviolette M (2002). Bronchial inflammation in corticosteroid-sensitive and corticosteroid-resistant asthma at baseline and on oral corticosteroid treatment. *Clinical and Experimental Allergy*.

[5] Sumi Y, Hamid Q (2007). Airway remodeling in asthma. *Allergology International*.

[6] Stone J, Hinks LJ, Beasley R, Holgate ST, Clayton BA (1989). Reduced selenium status of patients with asthma. *Clinical Science*.

[7] Flatt A, Pearce N, Thomson CD, Sears MR, Robinson MF, Beasley R (1990). Reduced selenium in asthmatic subjects in New Zealand. *Thorax*.

[8] Mahan DC, Kim YY (1996). Effect of inorganic or organic selenium at two dietary levels on reproductive performance and tissue selenium concentrations in first-parity gilts and their progeny. *Journal of Animal Science*.

[9] Mahan DC, Parrett NA (1996). Evaluating the efficacy of selenium-enriched yeast and sodium selenite on tissue selenium retention and serum glutathione peroxidase activity in grower and finisher swine. *Journal of Animal Science*.

[10] Gajčević Z, Kralik G, Has-Schön E, Pavić V (2009). Effects of organic selenium supplemented to layer diet on table egg freshness and selenium content. *Italian Journal of Animal Science*.

[11] KüÜkbay FZ, Yazlak H, Karaca I (2009). The effects of dietary organic or inorganic selenium in rainbow trout (*Oncorhynchus mykiss*) under crowding conditions. *Aquaculture Nutrition*.

[12] Taylor JB, Finley JW, Caton JS (2005). Effect of the chemical form of supranutritional selenium on selenium load and selenoprotein activities in virgin, pregnant, and lactating rats. *Journal of Animal Science*.

[13] Kadrabova J, Mad’aric A, Kovacikova Z, Podivinsky F, Ginter E, Gazdik F (1996). Selenium status is decreased in patients with intrinsic asthma. *Biological Trace Elemement Research*.

[14] Andreadis AA, Hazen SL, Comhair SAA, Erzurum SC (2003). Oxidative and nitrosative events in asthma. *Free Radical Biology and Medicine*.

[15] Henricks PAJ, Nijkamp FP (2001). Reactive oxygen species as mediators in asthma. *Pulmonary Pharmacology and Therapeutics*.

[16] Hasselmark L, Malmgren R, Zetterstrom O, Unge G (1993). Selenium supplementation in intrinsic asthma. *Allergy*.

[17] Gazdik F, Kadrabova J, Gazdikova K (2002). Decreased consumption of corticosteroids after selenium supplementation in corticoid-dependent asthmatics. *Bratislavske Lekarske Listy*.

[18] Stow JL, Ching Low P, Offenhäuser C, Sangermani D (2009). Cytokine secretion in macrophages and other cells: pathways and mediators. *Immunobiology*.

[19] Ugusman A, Zakaria Z, Kien Hui C, Megat Mohd Nordin NA (2011). *Piper sarmentosum* inhibits ICAM-1 and Nox4 gene expression in oxidative stress-induced human umbilical vein endothelial cells. *BMC Complementary and Alternative Medicine*.

